# Clozapine‐Associated Pulmonary Embolism: Continuation of Clozapine Therapy With Concurrent Anticoagulation

**DOI:** 10.1155/crps/6686502

**Published:** 2026-01-06

**Authors:** M. R. Jahangir, C. Heerema, J. Biedermann, S. Dieleman, N. H. Grootendorst-van Mil

**Affiliations:** ^1^ Department of Psychiatry, Erasmus MC, University Medical Center Rotterdam, P.O. Box 2040, 3000 CA, Rotterdam, Netherlands, erasmusmc.nl; ^2^ Department of Internal Medicine, Erasmus MC, University Medical Center Rotterdam, P.O. Box 2040, 3000 CA, Rotterdam, Netherlands, erasmusmc.nl

**Keywords:** anticoagulation, clozapine, pulmonary embolism

## Abstract

Clozapine, a gold standard for treatment‐resistant schizophrenia, is associated with a range of adverse effects, including the rare but serious risk of pulmonary embolism (PE). The management of such complications, particularly in the absence of clear guidelines for preventive anticoagulation, poses significant challenges. We present a case of a male (in his late 30s) with schizophrenia who developed recurrent thromboembolic events during clozapine therapy. Despite the occurrence of a second PE, clozapine therapy was continued successfully with concurrent anticoagulation. This case highlights the need for individualized treatment strategies and underscores the critical gap in evidence regarding preventive anticoagulation in patients with clozapine.

## 1. Introduction

When first‐line antipsychotic treatments prove ineffective, clozapine is considered the gold standard treatment for psychotic disorders due to its superior efficacy in reducing psychotic symptoms. However, clozapine use is associated with various adverse effects, including the rare but serious risk of pulmonary embolism (PE). The evidence for preventive anticoagulation in patients on clozapine is limited, making management of this complication particularly difficult. The exact pathophysiologic mechanism of PE as an adverse effect of clozapine is not fully understood, but several interrelated pathophysiological factors are thought to contribute such as drug‐induced hypercoagulability, reduced mobility from sedation and weight gain, metabolic disturbances, and inflammation. In the case presented, the patient had a history of thromboembolic events during earlier clozapine therapy, which complicates the decision‐making process regarding continued use of clozapine.

## 2. Case Presentation

Mr. X, a man in his late 30s with schizophrenia, was admitted to the psychiatric department of our academic hospital for analysis of cognitive and depressive symptoms. We also wanted to evaluate potential medication changes. Diagnosed with schizophrenia in 2017 following multiple psychotic episodes, he was treated with various medications. His treatment history includes aripiprazole, haloperidol, quetiapine, amisulpride, and clozapine, each with varying success and side effects. He used clozapine in 2022 and 2024, each time followed by deep vein thrombosis (DVT). He previously reported emotional flatness, suicidal ideation, concentration difficulties, and a somber mood with flat affect. Psychomotor retardation, including mild word‐finding difficulties, was also noted. The patient was not known to have any other medical conditions at that time.

During the first episode of DVT in 2022, the patient was in the titration phase of clozapine, although the exact dosage at that time could not be determined due to incomplete documentation. He was started on acenocoumarol following the diagnosis of DVT. Due to the DVT, clozapine was switched to amisulpride. This was also the reason acenocoumarol could eventually be discontinued.

Besides the use of clozapine, several transient risk factors were present. The patient was significantly immobilized at that time due to his psychotic and negative symptoms, his obesity, and the fact that he was an active smoker at that time. Additionally, the clinical picture was complicated by a likely underlying infection: the patient developed fever without an identifiable focus despite extensive diagnostic evaluation and was treated empirically with intravenous antibiotics for 5 days. Laboratory testing during that episode revealed a single positive result for lupus anticoagulant (LAC), though two subsequent tests were negative.

During the current admission in 2024, no psychotic features were observed, but cognitive complaints and negative symptoms persisted. Upon admission, the patient was taking amisulpride 375 mg once daily and biperiden 2 mg. He presented as flat, lacking initiative, and difficult to motivate, with occasional thoughts of euthanasia but a determination not to give up. Following consultation with the patient and his parents, clozapine was reintroduced with active monitoring. This included weekly leukocyte differentiation in accordance with hospital guidelines, as well as thrice‐weekly assessment of vital functions (respiratory rate, heart rate, blood pressure, and body temperature). In consultation with Hematology and Internal Medicine, preventive anticoagulation was deemed unnecessary, considering the patient’s increased activity level compared to his previous, sedentary state during the earlier thromboembolic event. In addition, for the current admission, the patient stopped smoking.

Eight days after the initiation of clozapine, with a dosage of 100 mg clozapine, the patient developed a subfebrile temperature, general malaise, and abdominal discomfort accompanied by constipation. Additionally, tachycardia was noted, prompting a cardiology consultation to rule out clozapine‐induced myocarditis. An electrocardiogram (ECG) was performed and showed no abnormalities suggestive of myocarditis. The symptoms resolved spontaneously without the need for specific intervention. Seventeen days after initiation of clozapine, with a dosage of 150 mg clozapine, the patient returned from leave early with a swollen right leg. His father recognized the presentation as similar to a previous episode of thrombosis. The patient’s blood test results showed an elevated D‐dimer level of 2.50 mg/L. The patient’s clozapine level was 565 µg/L, and the des‐clozapine level was 353 µg/L. At that time, the patient was still undergoing a cross‐taper from clozapine to amisulpride and was receiving 300 mg of amisulpride. Other laboratory results, such as infection parameters and renal function, were within in the normal ranges. A duplex ultrasound of the leg was performed and showed no evidence of DVT. A CT pulmonary angiogram was conducted and revealed segmental and subsegmental PEs bilaterally, with signs of right ventricular strain. The patient was transferred to the Coronary Care Unit (CCU) for close monitoring with continuous telemetry. Anticoagulation therapy was initiated with low‐molecular‐weight heparin (LMWH) and subsequently transitioned to a direct oral anticoagulant (DOAC), apixaban. After 2 days of clinical stabilization, the patient was transferred back to the psychiatric ward. After consultation with the patient and consultation with Internal Medicine, the patient continued with clozapine concurrently with anticoagulation.

Clozapine titration was continued, along with a gradual reduction of amisulpride. From this point onward, nursing staff observed a mild improvement in negative symptoms. The patient’s parents also noted subtle positive changes—he smiled more frequently and spoke less about somatic complaints. However, the patient himself reported no subjective improvement. Biperiden was discontinued, as extrapyramidal symptoms had resolved. The medication at discharge included famotidine 20 mg tablet twice a day, glycopyrronium drink 1 mg three times a day as needed, clozapine 350 mg tablet once a day (the clozapine blood level was 565 µg/L), macrogol/salts 1 sachet twice a day, and apixaban 5 mg tablet twice a day.

## 3. Discussion

This case report illustrates a complex clinical scenario in which a patient with schizophrenia developed PE during treatment with clozapine. It underscores the significant thromboembolic risks associated with antipsychotic medications, particularly clozapine, and highlights the multifactorial nature of these events.

The development of PE in this patient, despite active monitoring and an initial decision against preventive anticoagulation, illustrates the unpredictable nature of thrombotic events in patients treated with clozapine. Clozapine is known to be associated with an increased risk of venous thromboembolism (VTE), although the precise pathophysiological mechanisms remain incompletely understood [1]. Proposed contributing factors include sedation‐induced immobility, metabolic side effects, and potential direct effects on the coagulation cascade [2, 3]. Emerging evidence suggests that clozapine may also exert prothrombotic effects through immunological and hematological pathways. Notably, elevated levels of antiphospholipid antibodies have been observed in patients treated with clozapine. This is also one of the risk factors for PE [2]. Additionally, an in vitro study found that clozapine increased platelet adhesion and aggregation, which may increase the risk of thromboembolism [3].

In this case, following a multidisciplinary decision, including consultation with Internal Medicine, it was decided not to initiate prophylactic anticoagulation prior to starting clozapine. This decision was based on the assessment that the patient’s prior thrombotic event was likely attributable to transient risk factors such as immobilization and smoking, both of which were no longer present. At the time of clozapine initiation, the patient had ceased smoking and had become significantly more physically active, thereby mitigating those earlier risks and supporting the decision that prophylactic anticoagulation was not warranted.

It is noteworthy that during the initial episode of DVT, LAC was detected once, but subsequent testing on two occasions returned negative results. Although this does not fulfill the laboratory criteria for antiphospholipid syndrome (APS), it did raise diagnostic considerations in the context of the patient’s thrombotic history.

Patients with severe mental disorders carry a high burden of cardiovascular risk factors, including metabolic syndrome, dyslipidemia, obesity, and sedentary lifestyle [4]. Additionally, baseline abnormalities in hemostatic proteins, including reduced levels of tissue plasminogen activator (t‐PA) and protein S, have been observed. These alterations may not only predispose patients to thromboembolic events but could also contribute to the pathophysiology of schizophrenia itself. For instance, t‐PA facilitates the conversion of proBDNF to mature brain‐derived neurotrophic factor (BDNF), which supports synaptic plasticity and cognitive functioning. Reduced t‐PA activity may impair this conversion, potentially contributing to cognitive deficits [5]. Furthermore, t‐PA influences N‐methyl‐D‐aspartate (NMDA) receptor activity and dopamine signaling, further implicating it in the cognitive and negative symptoms seen in schizophrenia [5].

In our patient, negative symptoms and cognitive dysfunction were prominent, reflecting the complex interplay between psychiatric pathology, pharmacological treatment, and thrombotic risk. Increasing evidence suggests that disruptions in coagulation pathways—particularly involving t‐PA and protein S—may underlie both neurocognitive impairments and increased thrombotic susceptibility in schizophrenia.

This case also aligns with findings from a recent review of 42 case reports, which showed that 71.8% of VTE events occurred within the first 6 months of clozapine treatment, with a mean onset of 7.5 months [6]. Our patient developed PE on day 17, consistent with this high‐risk early treatment window. Ronaldson similarly reported that the risk of VTE doubled in the first 3 months of antipsychotic therapy compared to nonusers [7].

The dose of clozapine in our case (350 mg/day) falls within the range of doses reported at the time of VTE onset in the literature (50–700 mg/day), with a mean of 285.6 mg [6]. While some studies suggest a dose‐dependent relationship between clozapine dosage and VTE risk [8, 9], others found no such association [10, 11].

Polypharmacy is another relevant factor. In the reviewed cases, 60% of patients were on another antipsychotic at the time of VTE onset. Our patient was also receiving amisulpride, which was gradually tapered during clozapine titration. The contribution of polypharmacy to thrombotic risk remains difficult to isolate but should be considered in risk assessments.

Demographic factors such as age and sex also influence VTE risk. The mean age in reviewed cases was 43 years, with a male predominance. Our patient, a middle‐aged male, fit this profile. While some studies suggest higher VTE risk in elderly patients [12, 13], others found no significant age‐related difference [10].

Although the patient had a history of DVT, no other underlying risk factors (such as immobilization, malignancy, or inherited thrombophilia) were identified at the time of the second event. Importantly, both episodes occurred shortly after the reintroduction of clozapine, suggesting a temporal association. While causality cannot be established with certainty, the recurrence of DVT in close relation to clozapine initiation supports the attribution to clozapine as a contributing factor. Nevertheless, we acknowledge that other, as yet unidentified, mechanisms may also have played a role.

A systematic review [14] found that nearly half of the patients using clozapine who developed PE had no other risk factors besides clozapine use. Among these patients, anticoagulation therapy was chosen as the treatment in 80% of the cases, emphasizing its significance in managing this risk. In the same review, only 21 out of 33 patients with clozapine‐associated PE survived. Although this mortality rate is high, the overall mortality rate in patients with schizophrenia treated with clozapine remains lower than in those not treated with clozapine, given the drug’s effectiveness and the rarity of this side effect [15].

The presence of additional risk factors may warrant consideration of prophylactic strategies; however, there is currently insufficient evidence to support the routine use of prophylactic anticoagulation in clozapine‐treated patients. While clozapine has been associated with an increased risk of VTE, the existing literature does not provide robust data to justify preventive anticoagulation in the absence of identifiable risk factors.

Not much is known about continuing clozapine after surviving PE. Our patient continued clozapine while being treated with anticoagulation. In the aforementioned review, only four of the surviving patients continued clozapine treatment. In one case, clozapine was continued with no further episodes for 3 months while on anticoagulation. Another patient who continued clozapine stopped anticoagulation after 9 months, with no further episodes during a 2‐year follow‐up. One study reported two cases where clozapine was continued, with one patient on warfarin for 2 years without further PE episodes, although follow‐up data were limited.

Given the significant psychiatric benefits that clozapine offers, particularly for patients with treatment‐resistant schizophrenia, the decision to continue clozapine despite recurrent thromboembolic events was made in consultation with a multidisciplinary team. This decision underscores the importance of individualized care, where the risks and benefits of continuing treatment must be carefully weighed.

In this case, lifelong anticoagulation is indicated to mitigate the risk of recurrent thromboembolic events—regardless of whether clozapine is continued or discontinued. This decision is consistent with current clinical guidelines, which recommend indefinite anticoagulation after two episodes of VTE, even if one of those events was associated with a transient risk factor.

This approach reflects the current understanding that while preventive anticoagulation in clozapine‐treated patients is not routinely recommended due to the lack of robust evidence, it may be warranted in patients with a history of thromboembolism. The decision to continue clozapine with concurrent anticoagulation therapy highlights the necessity of balancing psychiatric stability with physical health risks. While anticoagulants are essential in the prevention and treatment of thromboembolic disorders, their use is not without significant risks. The most common and serious adverse effect is an increased risk of bleeding, which can range from minor bruising to life‐threatening hemorrhages [16].

The slight improvement in negative symptoms observed after continuation of clozapine, despite the ongoing risk management challenges, further illustrates the drug’s importance in managing severe psychiatric symptoms. However, this case also highlights the need for further research to establish clear guidelines on the use of anticoagulation in patients treated with clozapine to prevent thromboembolic events without compromising the mental health benefits.

## 4. Conclusion

This case underscores the challenges of managing thromboembolic risk in patients with schizophrenia treated with clozapine. Despite the occurrence of PE, clozapine was continued with concurrent lifelong anticoagulation, reflecting a careful balance between psychiatric benefit and physical risk. While clozapine is associated with increased VTE risk, current evidence does not support routine prophylactic anticoagulation in all patients. Individualized, multidisciplinary care remains essential, and further research is needed to guide anticoagulation strategies in this population.

## Conflicts of Interest

The authors declare no conflicts of interest.

## Author Contributions

M. R. Jahangir and C. Heerema contributed equally to this work and should be listed as cofirst authors.

## Funding

No funding was received for this manuscript.

## Data Availability

Data sharing is not applicable to this article as no datasets were generated or analyzed during the current study.
